# Plasmonic Chiral Metasurface Absorber Based on Bilayer Fourfold Twisted Semicircle Nanostructure at Optical Frequency

**DOI:** 10.1186/s11671-021-03474-6

**Published:** 2021-01-14

**Authors:** Yongzhi Cheng, Fu Chen, Hui Luo

**Affiliations:** 1 School of Information Science and Engineering, Wuhan University of Science and Technology, Wuhan, 430081 People’s Republic of China; 2 School of Optical and Electronic Information, Huazhong University of Science and Technology, 430074, Wuhan, People’s Republic of China

**Keywords:** Circular dichroism, Chiral selective absorption, Twisted-semicircle nanostructure, Plasmonic metasurface absorber

## Abstract

In this paper, we present a plasmonic chiral metasurface absorber (CMSA), which can achieve high selective absorption for right-handed and left-handed circular polarization (RCP, “+”, and LCP, “−”) lights at optical frequency. The CMSA is composed of a dielectric substrate sandwiched with bi-layer fourfold twisted semicircle metal nanostructure. The proposed CMSA has a strong selective absorption band, where absorption peaks for LCP and RCP lights occur at different resonance frequencies, reflecting the existence of a significant circular dichroism (CD) effect. It is shown that the absorbance of the CMSA can reach to 93.2% for LCP light and 91.6% for RCP light, and the maximum CD magnitude is up to 0.85 and 0.91 around 288.5 THz and 404 THz, respectively. The mechanism of the strong chiroptical response of the CMSA is illustrated by electric fields distributions of the unit-cell nanostructure. Furthermore, the influence of the geometry of the proposed CMSA on the circular polarization selective absorption characterization is studied systematically.

## Introduction

Chirality, as a ubiquitous phenomenon which refers to a geometric property of an object lacking any inversion or mirror plane symmetry, always remain relevant for science and technology [1, 2]. Chiral media usually appear in two enantiomeric forms which are mirror symmetric and non-superposable on its mirror image by simple translation or rotation, and they always exhibit different response to right-handed and left-handed circular polarization (RCP, “+”, and LCP, “−”) light [1]. Circular dichroism (CD) of circular polarization (CP) light originated from chiral media is one of the most unique chiroptical properties. The CD effect refers to different response of the RCP and LCP lights in chiral media which has wide application prospects in biology, medical science, chemistry, as well as polarization related optoelectronic devices [3–5]. However, the CD effect of natural materials is rather weak, which extremely hinders its practical application. Metasurfaces, as a sub-class of metamaterials consisting of a monolayer or few-layer planar structures, show great promise for arbitrary electromagnetic (EM) wave or light manipulation [6–10]. In particular, chiral metasurface (CMS) is able to enhance the chiral optical effects by several orders of magnitude.

The CMSs have received tremendous interest since it could exhibit exotic EM properties including negative refractive index and optical activity [11, 12], asymmetric transmission [13, 14], giant CD effect [15–17], polarization conversion [18, 19], and wave front manipulation [20, 21] etc. Since then, various CMSs structures (such as split-ring, spiral wire, gammadion, L-shaped and so on) have been successively proposed to achieve highly-efficient chiral-selective field enhancement for LCP or RCP light [22–33]. However, most of previous designs of those CMS focus on the performance of chirality in transmission, while much less attentions have been paid to the reflection/absorption for CP lights which are equally important in engineering applications. It is well known that the most studies of previous absorbers are applied to linear polarization light, whereas such designs for CP lights are rarely studied. In fact, the CMSs also could be used to construct novel absorbers for CP lights [25, 26, 29–33]. For instance, Li et al. [25] proposed an ultra-thin absorber based on the L-shaped wires, which can only attenuate the LCP wave in microwave region. Wang et al. [29] demonstrated that a chiral metamirror can almost reflect all the LCP light, while totally absorb the RCP light in infrared region. Tang et al. [30] proposed an absorber with ŋ-shaped-resonators, which can achieve selective absorption for different CP lights in visible. Then, near-infrared chiral absorbers with plasmonic metasurface have been proposed and demonstrated to selectively absorb LCP or RCP light. However, the absorbance of the most CMSs is less than 90%. Thus, an effective design of the chiral metasurface absorber (CMSA) with the high selective absorption is highly desirable.

In this work, we present one kind of highly-efficient CMSA based on bilayer fourfold twisted semicircle nanostructure working in near infrared and visible region. Such CMSA could selectively achieve over 90% absorption for different handed CP lights at different resonance frequency. Due to the strong selective absorption of the proposed CMSA, a high CD value of about 0.9 can be realized accordingly. The physical mechanism underlying in selective absorption for different CP lights has been analyzed in detail by electric field distributions. Furthermore, the influences of unit-cell geometric parameters towards selective absorption have been studied systematically as well. It can be reasonably believed that the results in this work can guide the design of CMSA with strong absorption and CD effect for many practical applications such as thermal absorber, optical communication devices, photodetector, optical filters, imaging and holograms.

## Unit-Cell Design

**Fig. 1:**
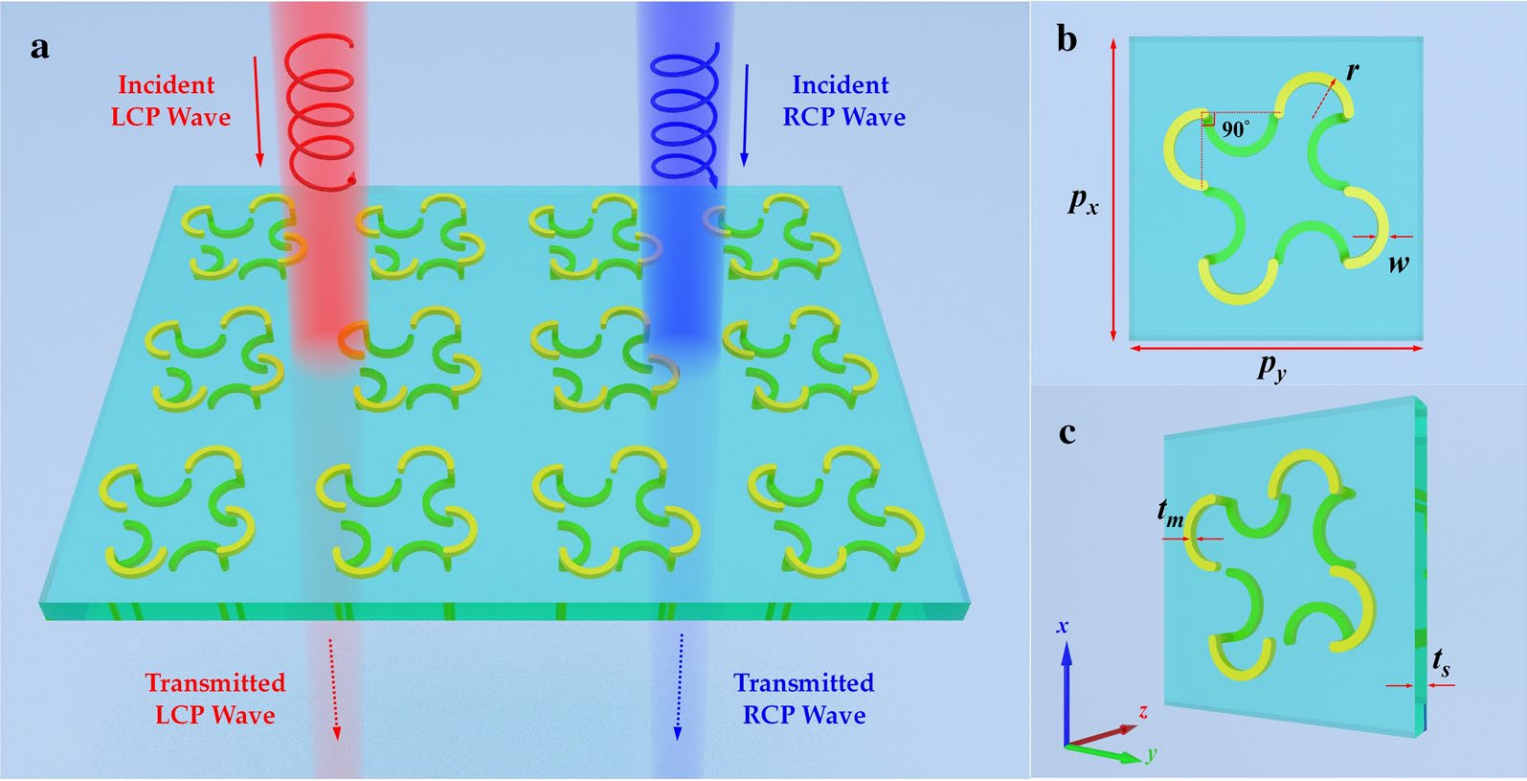
Schematic of the designed CMSA: **a** periodic array, **b**, **c** the front and perspective view of the unit-cell nanostructure. The periodic lengths along *x*- and *y-*axis directions are both 600 nm, and the normal incident CP lights are propagating along the *z*-axis direction

Figure 1 present the schematic diagram of the proposed CMSA, which is made of a periodic array with twisted semicircle nanostructure. The fourfold twisted semicircle nanostructure on each side of the dielectric substrate is positioned so that each one rotated by 90° with respect to its neighbor, and the bottom side each semicircle nanostructure is also rotated by 90° with respect to the top one, as shown in Fig. 1b. Similarly to previous design [32], the top four semicircle nanostructures are connected with the bottom one by copper cylinders, and the radius of copper cylinder is the same with the semicircle wire width, which can increase conductive coupling. The twisted semicircle nanostructure can be viewed as a coupled resonator system, where the strong chiral responses arise from the electric and magnetic inductive coupling between the two twisted connected semicircles [34, 35]. This simple twisted semicircle nanostructure with mirror symmetries are designed in the top and bottom layers allowing the proposed CMSA to enhance the chirality.

The overall unit-cell of the proposed CMSA exhibits a fourfold rotational (C_4_) symmetry for light propagation direction. The dielectric substrate in the middle layer is made of a loss-free dielectric MgF_2_ with relative permittivity of 1.9. The chiral metal nanostructures were selected as gold, and the material parameter can be described by the Drude model [36]. The optimized structure parameters of the unit-cell are given as: *p*_*x*_ = *p*_*y*_ = 600 nm, *r* = 70 nm, *w* = 40 nm, *t*_s_ = 120 nm, *t*_m_ = 30 nm. The unit-cell of the CMSA is periodic along the *x-* and *y*-axis directions with the periods of 600 nm to avoid diffraction when the incidence light frequency comes up to 500 THz. To verify the efficiency of the proposed CMSA, the full-wave high frequency EM simulations were performed based on the finite element method (FEM) by using the frequency domain solver in CST Microwave Studio. Once the CMSA unit-cell nanostructure, proper boundary conditions, mesh size and frequency range have been reasonably set, the frequency domain simulation could be launched.

## Results and Discussions

**Fig. 2:**
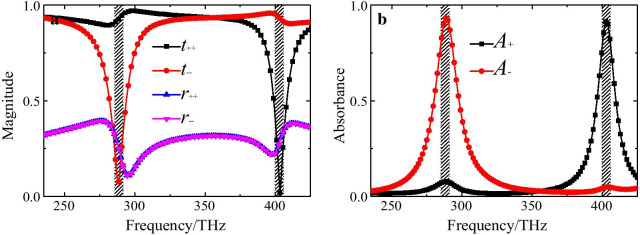
**a** Simulated co-polarization transmission coefficients (*t*_++_(ω), *t*_− −_(ω)) and reflection coefficients (*r*_++_(ω), *r*_− −_(ω)) of the proposed CMSA for the normal incident LCP and RCP lights, **b** the corresponding absorbance (*A*_+_(ω), *A*_−_(ω)) for both LCP and RCP lights

The simulated co-polarization transmission coefficients (*t*_++_(ω), *t*_− −_(ω)) and reflection coefficients (*r*_++_(ω), *r*_− −_(ω)) for normal incident LCP and RCP lights are presented in Fig. 2. Two chiral plasmonic resonance modes (mode 1 and mode 2) could be evidently observed at frequencies of *f*_1_ = 288.5 THz and *f*_2_ = 404 THz, respectively. It can be observed that the co-polarization reflection coefficient *r*_++_(ω) for RCP and *r*_− −_(ω) for LCP lights are equal; and both of them are less than 0.4 in the whole interested frequency range. In addition, the magnitudes of *r*_++_(ω) and *r*_− −_(ω) decrease to about 0.15 at resonances, indicating the impedance-matching between the CMSA and free space for both RCP and LCP lights. It can be also seen that the co-polarization transmission coefficients *t*_++_(ω) for RCP and *t*_− −_(ω) for LCP lights are significant different at resonances due to the chiral nature of the proposed CMSA. Around the lower frequency point *f*_1_, the magnitude of *t*_++_(ω) for the RCP light is about 0.93, which is much higher than *t*_− −_(ω) for the LCP light which is about only 0.075. Around the higher frequency point *f*_2_, the magnitude of *t*_++_(ω) for the RCP light decreases to minimal value of 0.018, while *t*_− −_(ω) for the LCP is up to the maximal value of about 0.92. It means that only the incident RCP light can be selected to pass through the CMSA while the LCP light is forbidden at the lower frequency. As at the higher frequency *f*_2_, only the incident LCP light can be selected to pass through the CMSA while the RCP light is forbidden extremely. Thus, the chiral selection phenomena of the CMSA above would consequently result in the different absorption for the RCP and LCP lights, implying the existence of a high efficiency selective absorption and giant CD effect at resonances.

Figure 2b shows the absorbance spectra (*A*_+_(ω), *A*_−_(ω)) for both incident LCP and RCP lights. It can be observed that the absorbance for LCP and RCP lights is up to maximal value of about 93.2% and 91.6%, while the one for RCP and LCP lights is decreased to only about 8.7% and 4.8% around the above two resonances, respectively. Obviously, it can be concluded that the proposed CMSA exhibits a strong absorption of LCP light and a rather high transmission level for the RCP light around the lower frequency *f*_1_, whereas the condition completely reverses when the frequency comes up to the higher resonance frequency *f*_2_. It means that the proposed CMSA displays a selective absorption for two CP lights with particular handedness while reflecting the other one at different resonances. Besides, it is also worth highlighting that the CMSA has the two strong absorption frequency band just using one single size chiral nanostructure, and is reasonably superior compared with the previous chiral absorbers with one isolated absorption band whose adaption for different CP light is highly dependent on different geometry size [25, 26, 29, 31–33]. Thus, the designed chiral nanostructure can act serve as a perfect LCP light absorber at the lower frequency and perfect RCP light absorber at the higher frequency. It should be noticed that the selective absorption performance of the proposed CMSA will deteriorate with the increase of the incident angle (oblique incidence), due to the higher-order multipolar plasmon resonance (not shown). Furthermore, it can be inferred that the high chiral-selective absorption for CP lights will result in a giant CD effect in proposed CMSA.

**Fig. 3:**
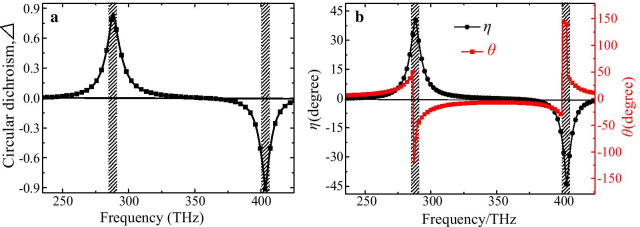
The calculated relative optical parameters of the proposed CMSA for the normal incident LCP and RCP lights, **a** the CD parameter *Δ*, **b** ellipticity angle *η* and polarization azimuth rotation angle *θ*

The difference of absorption or transmission between the LCP and RCP lights can be characterized by CD parameter *Δ*. Figure 3a presents the CD spectrum of the CMSA, where the main peaks of CD parameter are about 0.85 and 0.91 at two resonance frequencies, respectively; which is much greater than the reported chiral nanostructures [17–32, 37–42]. The giant CD effect is caused by the strong chirality of the CMSA, and therefore can be applied as a transparent circular polarizer. To further investigate the CP purity of the CMSA applied as a circular polarizer, we give the ellipticity angle *η* and polarization azimuth rotation angle *θ* as shown in Fig. 3b. It can be found that the value of *η* is about 40.4° and − 43.9°, while the value of *θ* is about 0° at the lower and higher frequencies, respectively. It means that the transmitted lights exhibit prominent RCP and LCP characteristics after passing through the CMSA slab at the two resonance frequencies. It should be noticed that this CMSA-based circular polarizer with the higher CP purity is valid for any arbitrarily polarization lights due to its high C_4_ symmetry of the unit-cell. Thus, the homogenous circular polarizer could be reasonably believed to be realized with our designed chiral nanostructure.

**Fig. 4:**
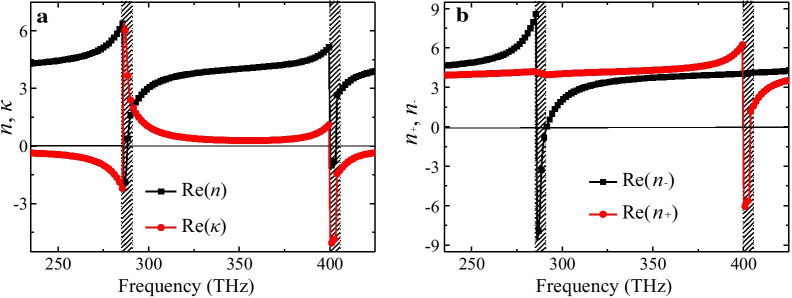
The retrieved relative chiral parameters of the proposed CMSA: **a** real parts of average refractive index Re(*n*) and chiral parameter Re(*κ*), **b** refractive index Re(*n*_−_), Re(*n*_+_) for LCP and RCP lights

In order to fully understand the selective absorption and giant CD effect of the CMSA, we retrieved the refractive index Re(*n*), Re(*n*_−_), Re(*n*_+_) and chiral parameter Re(*κ*) using a standard retrieval procedure from the transmission and reflection coefficients of CP lights [43, 44], as shwon in Fig. 4a, b. It is clear that two resonances related to the strong chirality emerge in the designed CMSA. The lower frequency resonance occurs around 288.5 THz while the higher one locates at 404 THz, which are consistent with the characteristic frequecies of selective absorption and CD peaks. As shown in Fig. 4a, the Re(*n*) is negative with maximal magnitudes of − 2.3 and − 1.1, and the Re(*κ*) is up to maximal magnitudes of 6.4 and − 5.1 around the two resonance frequencies above. It is clear that the chiral parameter *κ* also contributes to the negative refraction of RCP and LCP lights. The strong chirality can easily push the refractive index of the RCP/LCP light to become negative at resonances due to the relation of *n*_±_ = *n* ± *κ*. Thus, as shown in Fig. 4b, the Re(*n*_−_) for LCP light and Re(*n*_+_) for RCP light is negative from 286.2 THz to 291 THz, and 400.2 THz to 404 THz, respectively. In addition, the Re(*n*_−_) and Re(*n*_+_) are up to the maximal negative values of − 8.6 and − 6.3 at two resonances above, respectively. It reveals that the high selective absorption as well as the giant CD effect of the proposed CMSA are associated with the negative refractive property of the LCP and RCP lights.

To further unveil the origin of the selective absorption associated with the giant CD effect of the proposed CMSA, the electric field (*E*_z_) distributions of the unit-cell driven by RCP and LCP lights at 288.5 THz and 404 THz has been studied. It has been known that the excitation of surface plasmons resonance will produce oscillating dipole fields since the semicircle nanostructure exhibits small diameters compared with the incident wavelength of the different CP lights [45–48]. When RCP or LCP light illuminate onto the semicircle nanostructure, it can be reasonably believed that the selective absorption and giant CD effect will emerge in the proposed CMSA and consequently resutling in the different distribution of the electric field and magnetic field components in each layer [48–53].

**Fig. 5:**
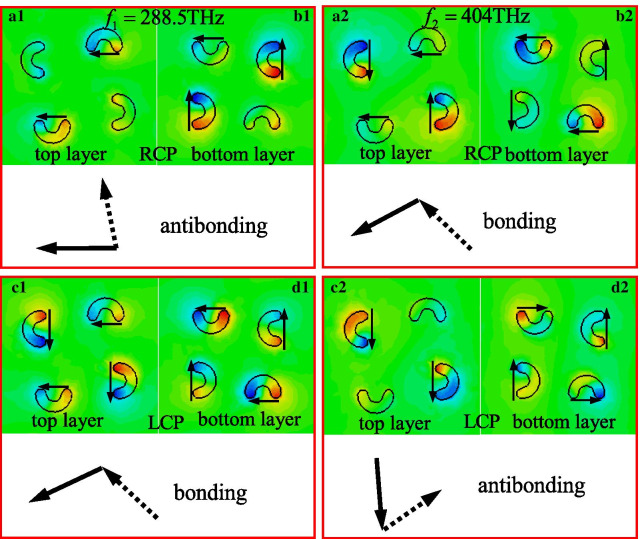
The electric field (*E*_z_) distributions of unit-cell of the proposed CMSA induced by the (**a1**, **b1**, **a2**, **b2**) RCP and (**c1**, **d1**, **c2**, **d2**) LCP lights at different resonance frequencies: (**a1**–**d1**) *f*_1_ = 288.5 THz, (**a2**–**d2**) *f*_2_ = 404 THz. The black solid line (dash line) arrows denote the equivalent electric dipole moments on the top (bottom) layer of the proposed chiral nanostructure

Figure 5 show the electric field (*E*_z_) distributions of the proposed CMSA drived by RCP and LCP lights at different resonance frequencies. The detail plots of the electric field (*E*_z_) distributions on the semicircle nanostructures clearly show the nature of each sruface plasmonics mode [54]. The red and blue region on the semicircle nanostructure of the top and bottom layers present the positive and negative charge accumulations under RCP and LCP light excitation. The positive and negative charges are separated and mainly accumulated at the corners of the each semicircle nanostructure, acting like an electric dipole oscillation. It can be observed that the electric dipole power is much stronger than the magnetic one in the designed semicircle nanostructure, revealing the predominance of electric dipole oscillations. The selective absorption and giant CD effect generated at resonances is owing to the obvious dipole power different under LCP and RCP excitation. Here, a simplified method with equivalent electric dipole moments has been applied, which considers the charge vibrations of four semicircle nanostructure in each layer as one dipole vibration [48–50]. According to Born-Kuhn theory [47, 48], the mode which is hybridized from two dipoles with the same electric field direction is named as bonding mode, while the other one which is hybridized from two dipoles with 90° or cross direction is denoted as antibonding mode. As shown in Fig. 5a1, b1, under RCP light illumination at resonance frequency of *f*_1_ = 288.5 THz, the electric dipole fields in top and bottom layers shows the cross directions and form an antibonding mode, and consequently resulting in the high transmission of RCP light according to Born-Kuhn model. As shown in Fig. 5c1, d1, under LCP light illumination, it can be seen that the electric field distribution of CMSA can be considered to be a hybrid from the bonding mode between the upper and lower layers, which is composed of two equivalent electric dipole moments with the same direction, resulting in high absorption level of LCP light. Thus, the bonding and antibonding modes cause different resonance energy and different transmission and absorption of chiral nanostructures at the lower frequency under LCP and RCP lights illumination (see Fig. 2). As shown in Fig. 5a2, b2, c2, d2, under RCP and LCP lights illumination at resonance frequency of *f*_2_ = 404 THz, the electric dipole fields in top and bottom layers shows the same directions (bonding mode) and cross directions (antibonding mode) respectively, and consequently resulting in high absorption level for RCP light and high transmission for LCP light. Hence, it can be seen that the selective absorption and CD effect at two different frequencies are mainly attributed to the bonding and antibonding modes, which is induced by hybrid coupling of the top and bottom layer electric dipole moments.

**Fig. 6:**
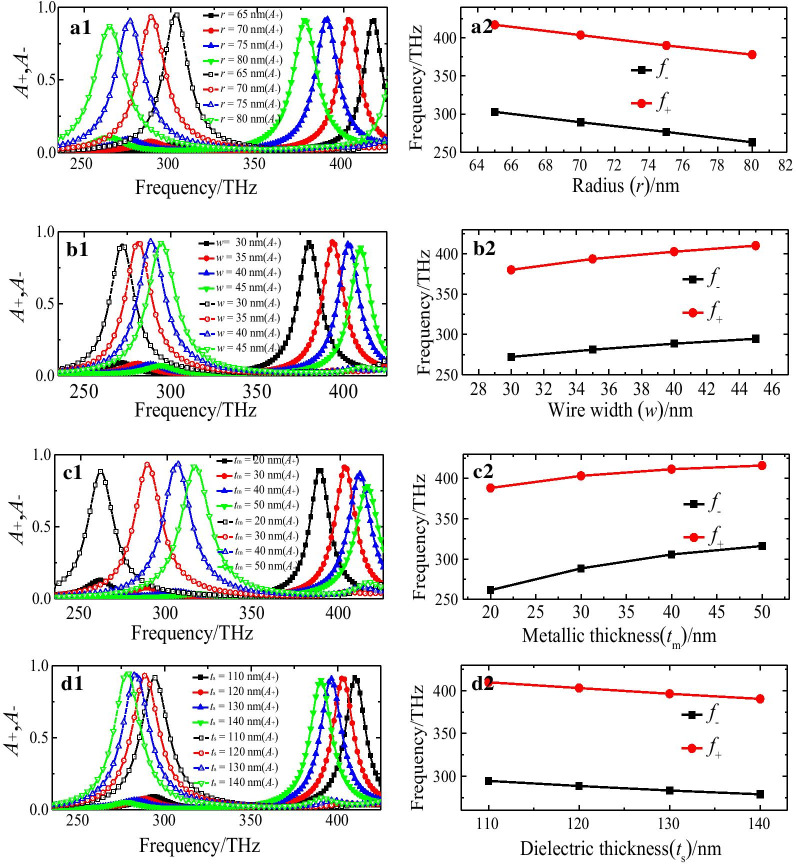
The simulated (**a1**–**d1**) absorbance spectra and (**a2**–**d2**) resonance frequencies (*f*_−_, *f*_+_) of the LCP and RCP lights of proposed CMSA with different geometric parameters: (**a1**, **a2**) radius (*r*), (**b1**, **b2**) wire width (*w*), and (**c1**, **c2**) thickness (*t*_m_) of semicircle nanostructure, (**d1**, **d2**) thickness of dielectric substrate (*t*_s_)

In the following, we investigate the influences of the geometric parameters of the unit-cell on the absorption properties of the proposed CMSA. Figure 6 shows the simulated absorbance spectra for LCP and RCP lights, and resonance frequencies (*f*_−_, *f*_+_) with different geometric parameters (*r*, *w*, *t*_m_, and *t*_s_) of the unit-cell. For the designed nanostructure, some interesting spectral variation of parameter-depended selective absorption property, which is obviously multi-factors competitive and complex, could be observed. In this study, the geometric parameters of the control group are *r* = 70 nm, *w* = 40 nm, *t*_m_ = 30 nm, and *t*_s_ = 120 nm, and changing one parameter at a time.

The semicircle nanostructure with the different *r* (*r* = 65 nm, 70 nm, 75 nm, and 80 nm) were firstly calculated, while the other parameters are fixed as shown in Fig. 6a1, a2. When increases *r*, the resonance frequencies *f*_−_ for LCP and *f*_+_ for RCP lights decrease gradually, which can be interpreted by the equivalent *LC* resonance circuit theory [55, 56]. The resonance frequencies (*f*_−_, *f*_+_) for both LCP and RCP lights illumination can be expressed as $f_{ \mp } = \frac{1}{{2\pi \sqrt {LC} }}$, where equivalent capacitance *C* and inductance *L* are mainly determined by the geometric parameters of the proposed CMSA. The *L* will increase with the increase of the *r*, thus resulting in the decrease of the resonance frequencies (*f*_−_, *f*_+_). In addition, as shown in Fig. 6a1, when increases *r*, the absorbance of the LCP light will decrease gradually while the one of the RCP light will be nearly unchanged. Figure 6b1, b2 shows the absorbance spectra of the LCP and RCP lights when changing the *w* from 30 to 45 nm by a step of 5 nm, while the other parameters are kept unchanged. It can be seen that the resonance frequencies (*f*_−_, *f*_+_) for both LCP and RCP lights will increase gradually with the increase of the *w*. Obviously, the increase of the resonance frequencies (*f*_−_, *f*_+_) is mainly due to the decrease of the *C*. The absorbance of the LCP light will firstly increase and then decrease slightly, while the one of the RCP light will decrease gradually when increasing *w*, as shown in Fig. 6b2. As shown in Fig. 6c1, c2, we present the absorbance spectra and resonance frequencies (*f*_−_, *f*_+_) of the LCP and RCP lights with varying *t*_m_ from 20 to 50 nm by a step of 10 nm and other parameters fixed. There are similar cases to the change of *w*, when increases *t*_m_, the resonance frequency (*f*_−_) for LCP light increase significantly, and the one for RCP light increase slightly. In this case, the *L* will decrease with the increasing of the *t*_m_, thus resulting in the increase of the resonance frequencies (*f*_−_, *f*_+_). In addition, the absorbance of both LCP and RCP lights will firstly increase and then decrease when increasing *t*_m_, as shown in Fig. 6c2. Finally, we illustrated the absorbance spectra and resonance frequencies (*f*_−_, *f*_+_) for both LCP and RCP lights with different *t*_s_ (*t*_s_ = 110 nm, 120 nm, 130 nm, and 140 nm), while other parameters are kept unchanged, as shown in Fig. 6d1, d2. It can be observed that when increases *t*_*s*_, the absorbance of the LCP will increase gradually, while the one of the RCP light will decrease slightly, as shown in Fig. 6d1. In addition, the resonance frequencies (*f*_−_, *f*_+_) for both LCP and RCP lights decrease gradually when increases *t*_*s*_, as shown in Fig. 6d2. In this case, the *C* will increase when increases *t*_*s*_, thus resulting in the decrease of the resonance frequencies (*f*_−_, *f*_+_). It can be concluded that the resonance frequencies (*f*_−_, *f*_+_) and absorption level for both RCP and LCP lights are sensitive to the geometric parameters of the unit-cell of the designed chiral nanostructure. Thus, the selective absorption properties of the proposed CMSA can be adjusted dynamically by varying structure parameters.

## Conclusion

In conclusion, a CMSA based on a bi-layered fourfold twisted semicircle nanostructure has been proposed to achieve near perfect chiral-selective absorption for RCP and LCP lights as well as giant CD effect in both near-infrared and visible regions. The simulation results exhibit that the chiral-selective absorbance for both RCP and LCP lights are more than 90%, and the CD magnitude could reach to 0.91. According to the retrieved effective EM parameters, it can be found that the lower frequency absorption and CD effect are both associated with the negative refraction properties of the LCP light, whereas the case of higher frequency is concerned with the RCP light. The electric field distributions indicate that the chiral-selective absorption properties and giant CD effect of the CMSA mainly originate from the bonding and antibonding modes which are induced by hybrid coupling of the top and bottom layer electric dipole moments. Furthermore, the resonance frequencies and chiral-selective absorption level of the CMSA can be tuned by changing the geometric parameters of the unit-cell. Hence, it can be reasonably concluded that the design of the CMSA is promising for future applications in optical filter, chiral imaging, circular polarizer, detecting, and optical communications.

## Numerical Method Section

FEM Simulations: Full wave EM simulations have been performed based on a finite element method (FEM). In simulation, the electrical properties of the gold are described by the Drude model as [36]:

1 $$\varepsilon_{{{\text{Au}}}} = {1} - \omega_{p}^{{2}} /\omega \left( {\omega + i\gamma } \right)$$

where *ω*_*p*_ = 1.37 × 10^16^ rad/s is the plasma frequency and *γ* = 8.04 × 10^13^ rad/s is collision frequency of the gold at optical frequency range. In simulation, the unit-cell boundary condition was applied along *x*- and *y*-axis direction and the two CP eigen lights were used directly. Broadband CP lights are employed as the excitation sources and are normally through the unit-cell of the designed chiral nanostructure from the − *z* to + *z* direction. Then, reflection and transmission coefficients of both LCP and RCP lights can be obtained. Generally, the absorbance denoted as *A*_−_(ω)/*A*_+_(ω) for the LCP/RCP lights can be expressed as [17, 32]: *A*_−_(ω) = 1 − *R*_− −_(ω) − *T*_− −_(ω) = 1 − |*r*_− −_(ω)|^2^ − |*t*_− −_(ω)|^2^, *A*_+_(ω) = 1 − *R*_++_(ω) − *T*_++_(ω) = 1 − |*r*_++_(ω)|^2^ − |*t*_++_(ω)|^2^, respectively. The co-polarization transmission coefficients *t*_− −_(ω) are for the LCP and *t*_++_(ω) for the RCP lights, while *r*_− −_(ω) and *r*_++_(ω) are the co-polarization reflection coefficients, respectively. It should be noticed that the cross-polarization transmission coefficients (*t*_+−_(ω), *t*_−+_(ω)) and reflection coefficients (*r*_+−_(ω), *r*_−+_(ω)) for LCP and RCP lights are small enough to be neglected (< 0.01) due to the high *C*_4_ symmetry of the unit-cell of the designed chiral nanostructure. In addition, the CD effect is induced by the selective absorption of two CP lights, which can be expressed as: *△* =|*t*_++_(ω)| − |*t*_− −_(ω)| [14, 29]. The ellipticity and optical activity are important parameters to evaluate the chirality of the designed chiral nanostructure. The ellipticity characterizes the polarization state of transmitted lights of the chiral nanostructure, which is described by ellipticity angle *η* = arctan[(|*t*_++_(ω)| − |*t*_− −_(ω)|) / (|*t*_++_(ω)| +|*t*_− −_(ω)|)]. While the optical activity represents the rotation property of polarization plane of a transmitted linear polarization light respect to the incident one, which is described by the polarization azimuth rotation angle *θ* = [arg(*t*_++_(ω))  − arg(*t*_− −_(ω))]/2.

## Data Availability

The datasets generated and/or analyzed during the current study are available from the corresponding author on reasonable request.

## Abbreviations

CMS: Chiral metasurface
CMSA: Chiral metasurface absorber
RCP: Right-handed circular polarization
LCP: Left-handed circular polarization
CD: Circular dichroism
EM: Electromagnetic
CP: Circular polarization

## References

[1] Barron LD (2004). Molecular light scattering and optical activity.

[2] Plum E, Zheludev NI (2015). Chiral mirrors. Appl Phys Lett.

[3] Yannopapas V (2009). Circular dichroism in planar nonchiral plasmonic metamaterials. Opt Lett.

[4] Wang B, Zhou J, Koschny T, Soukoulis C (2009). Nonplanar chiral metamaterials with negative index. Appl Phys Lett.

[5] Sharma V, Crne M, Park J, Srinivasarao M (2009). Structural origin of circularly polarized iridescence in jeweled beetles. Science.

[6] Cheng H, Liu Z, Chen S, Tian J (2015). Emergent functionality and controllability in few-layer metasurfaces. Adv Mater.

[7] He X, Lin F, Liu F, Zhang H (2020). Investigation of phonon scattering on the tunable mechanisms of terahertz graphene metamaterials. Nanomaterials.

[8] Peng J, He X, Shi C, Leng J, Lin F, Liu F, Zhang H, Shi W (2020). Investigation of graphene supported terahertz elliptical metamaterials. Physica E.

[9] He X, Lin F, Liu F, Shi W (2020). Tunable strontium titanate terahertz all-dielectric metamaterials. J Phys D Appl Phys.

[10] He Q, Sun S, Xiao S, Zhou L (2018). High-effiiency metasurfaces: principles, realizations, and applications. Adv Opt Mater.

[11] Cole M, Chen W, Liu M, Kruk S, Padilla W, Shadrivov I, Powell D (2017). Strong broadband terahertz optical activity through control of the blaschke phase with chiral metasurfaces. Phys Rev Appl.

[12] Chen H, Cheng Y, Zhao J, Mao X (2018). Multi-band terahertz chiral metasurface with giant optical activities and negative refractive index based on T-shaped resonators. Mod Phys Lett B.

[13] Fedotov V, Schwanecke A, Zheludev N, Khardikov V, Prosvirnin S (2007). Asymmetric transmission of light and enantiomerically sensitive plasmon resonance in planar chiral nanostructures. Nano Lett.

[14] Liu D, Xiao Z, Wang Z (2017). Multi-band asymmetric transmission and 90° polarization rotator based on bi-Layered metasurface with F-shaped structure. Plasmonics.

[15] Wang Z, Teh B, Wang Y, Adamo G, Teng J, Sun H (2017). Enhancing circular dichroism by super chiral hot spots from a chiral metasurface with apexes. Appl Phys Lett.

[16] Zhang M, Lu Q, Zheng H (2018). Tunable circular dichroism created by surface plasmons in bilayer twisted tetramer nanostructure arrays. J Opt Soc Am B.

[17] Cheng Y, Chen F, Luo H (2020). Multi-band giant circular dichroism based on conjugated bilayer twisted-semicircle nanostructure at optical frequency. Phys Lett A.

[18] Wu S, Xu S, Zinenko T, Yachin V, Prosvirnin S, Tuz V (2019). 3D-printed chiral metasurface as a dichroic dual-band polarization converter. Opt Lett.

[19] Cheng Y, Fan J, Luo H, Chen F (2020). Dual-band and high-efficiency circular polarization convertor based on anisotropic metamaterial. IEEE Access.

[20] Li Z, Cai X, Tian Y, Liu J (2019). Shaping the wavefront of light with high-order and short-period mode metasurfaces. Opt Commun.

[21] Fan J, Cheng Y (2020). Broadband high-efficiency cross-polarization conversion and multi-functional wavefront manipulation based on chiral structure metasurface for terahertz wave. J Phys D Appl Phys.

[22] Zhao R, Zhang L, Zhou J, Koschny T, Soukoulis CM (2011). Conjugated gammadion chiral metamaterial with uniaxial optical activity and negative refractive index. Phys Rev B.

[23] Ma X, Huang C, Pu M, Hu C, Feng Q, Luo X (2012). Multi-band circular polarizer using planar spiral metamaterial structure. Opt Express.

[24] Cheng Y, Nie Y, Wu L, Gong R (2013). Giant circular dichroism and negative refractive index of chiral metamaterial based on split-ring resonators. Prog Electromagn Res.

[25] Li M, Guo L, Dong J, Yang H (2014). An ultra-thin chiral metamaterial absorber with high selectivity for LCP and RCP waves. J Phys D.

[26] Li W, Coppens Z, Besteiro L, Wang W, Govorov A, Valentine J (2015). Circularly polarized light detection with hot electrons in chiral plasmonic metamaterials. Nat Commun.

[27] Shang X, Zhai X, Wang L, He M, Li Q, Luo X, Duan H (2017). Asymmetric transmission and polarization conversion of linearly polarized waves with bilayer L-shaped metasurfaces. Appl Phys Express.

[28] Chen Y, Gao J, Yang X (2018). Chiral metamaterials of plasmonic slanted nanoapertures with symmetry breaking. Nano Lett.

[29] Wang Z, Jia H, Yao K, Cai W, Chen H, Liu Y (2016). Circular dichroism metamirrors with near-perfect extinction. ACS Photonics.

[30] Tang B, Li Z, Palacios E, Liu Z, Butun S, Aydin K (2017). Chiral-selective plasmonic metasurface absorbers operating at visible frequencies. IEEE Photonics Technol Lett.

[31] Ouyang L, Wang W, Rosenmann D, Czaplewski D, Gao J, Yang X (2018). Near-infrared chiral plasmonic metasurface absorbers. Opt Express.

[32] Cheng Y, Chen H, Zhao J, Mao X, Cheng Z (2018). Chiral metamaterial absorber with high selectivity for terahertz circular polarization waves. Opt Mater Express.

[33] Kong X, Khorashad L, Wang Z, Govorov A (2018). Photothermal circular dichroism induced by plasmon resonances in chiral metamaterial absorbers and bolometers. Nano Lett.

[34] Cui Y, Kang L, Lan S, Rodrigues S, Cai W (2014). Giant chiral optical response from a twisted-arc metamaterial. Nano Lett.

[35] Sun B, Yu Y (2018). Analysis of circular dichroism in chiral metamaterial at terahertz frequencies. J Phys D Appl Phys.

[36] Ordal M, Long L, Bell R, Bell S, Bell R, Alexander W, Ward C (1983). Optical properties of the metals Al Co, Cu, Au, Fe, Pb, Ni, Pd, Pt, Ag, Ti, and W in the infrared and far infrared. Appl Opt.

[37] Hu L, Tian X, Huang Y, Fang L, Fang Y (2016). Quantitatively analyzing the mechanism of giant circular dichroism in extrinsic plasmonic chiral nanostructures by tracking the interplay of electric and magnetic dipoles. Nanoscale.

[38] Wang T, Fu T, Chen Y, Zhang Z (2017). Circular dichroism of a tilted U-shaped nanostructure. Opt Lett.

[39] Wang F, Fu T, Wang Y, Zhang Y, Zhang Z, Wang L (2018). Plasmonic circular dichroism of a tailed spatial cross-shaped nanostructure. J Opt Technol.

[40] Feng X, Bai Y, Jing Z, Qu Y, Wang T, Ullah H, Zhang Z (2019). Enhanced circular dichroism of tilted zigzag-shaped nanohole arrays. Appl Opt.

[41] Qi J, Zhang M, Zhang Y, Han Q, Gao W, Wang Y, Miao R, Dong J (2019). Multiband circular dichroism from bilayer rotational F4 nanostructure arrays. Appl Opt.

[42] Rajaei M, Zeng J, Albooyeh M, Kamandi M, Hanifeh M, Capolino F, Wickramasinghe H (2019). Giant circular dichroism at visible frequencies enabled by plasmonic ramp-shaped nanostructures. ACS Photonics.

[43] Zhou J, Dong J, Wang B, Koschny T, Kafesaki M, Soukoulis C (2009). Negative refractive index due to chirality. Phys Rev B.

[44] Zhao R, Koschny T, Soukoulis C (2010). Chiral metamaterials: retrieval of the effective parameters with and without substrate. Opt Express.

[45] Bohren C, Huffman D (1983). Absorption and scattering of light by small particles.

[46] Liu N, Kaiser S, Giessen H (2008). Magnetoinductive and electroinductive coupling in plasmonic metamaterial molecules. Adv Mater.

[47] Wang Y, Qin F, Yi Z, Chen X, Zhou Z, Yang H, Liao X, Tang Y, Yao W, Yi Y (2019). Effect of slit width on surface plasmon resonance. Results Phys.

[48] Hor Y, Phua W, Khoo E (2017). Chirality switching via rotation of bilayer fourfold meta-structure. Plasmonics.

[49] Liu N, Giessen H (2010). Coupling effects in optical metamaterials. Angew Chem Int Ed.

[50] Yin X, Schäferling M, Metzger B, Giessen H (2013). Interpreting chiral nanophotonic spectra: the plasmonic Born-Kuhn model. Nano Lett.

[51] Pan M, Su Z, Yu Z, Wu P, Jile H, Yi Z, Chen Z (2020). A narrowband perfect absorber with high Q-factor and its application in sensing in the visible region. Results Phys.

[52] Qin F, Chen Z, Chen X, Yi Z, Yao W, Duan T, Wu P, Yang H, Li G, Yi Y (2020). A tunable triple-band near-infrared metamaterial absorber based on Au nano-cuboids array. Nanomaterials.

[53] Luo J, Cheng Y, Gong Z, Wu K, Zhou Y, Chen H, Gauthier M, Cheng Y, Liang J, Zou T (2020). Self-assembled peptide functionalized gold nanopolyhedrons with excellent chiral optical properties. Langmuir.

[54] Yu P, Yang H, Chen X, Yi Z, Yao W, Chen J, Yi Y, Wu P (2020). Ultra-wideband solar absorber based on refractory titanium metal. Renew Energy.

[55] Maeda M (1972). An analysis of gap in microstrip transmission lines. IEEE Trans Microw Theory Technol.

[56] Li W, Cheng Y (2020). Dual-band tunable terahertz perfect metamaterial absorber based on strontium titanate (STO) resonator structure. Opt Commun.

